# Honokiol Eliminates Human Oral Cancer Stem-Like Cells Accompanied with Suppression of Wnt/****β****-Catenin Signaling and Apoptosis Induction

**DOI:** 10.1155/2013/146136

**Published:** 2013-04-10

**Authors:** Chih-Jung Yao, Gi-Ming Lai, Chi-Tai Yeh, Ming-Tang Lai, Ping-Hsiao Shih, Wan-Ju Chao, Jacqueline Whang-Peng, Shuang-En Chuang, Tung-Yuan Lai

**Affiliations:** ^1^Cancer Center, Wan Fang Hospital, Taipei Medical University, Taipei 11696, Taiwan; ^2^Center of Excellence for Cancer Research, Taipei Medical University, Taipei 11031, Taiwan; ^3^National Institute of Cancer Research, National Health Research Institutes, Miaoli 35053, Taiwan; ^4^Department of Surgery, Shuang Ho Hospital, Taipei Medical University, Taipei 23561, Taiwan; ^5^Graduate Institute of Clinical Medicine, Taipei Medical University, Taipei 11031, Taiwan; ^6^Graduate Institute of Medical Sciences, National Defense Medical Center, Taipei 11490, Taiwan; ^7^Department of Otolaryngology, Wan Fang Hospital, Taipei Medical University, Taipei 11696, Taiwan; ^8^Department of Traditional Medicine, Wan Fang Hospital, Taipei Medical University, Taipei 11696, Taiwan; ^9^Graduate Institute of Pharmacognosy, College of Pharmacy, Taipei Medical University, No. 250, Wu-Hsing Street, Taipei 11031, Taiwan

## Abstract

Honokiol, an active compound of *Magnolia officinalis*, exerted many anticancer effects on various types of cancer cells. We explored its effects on the elimination of cancer stem-like side population (SP) cells in human oral squamous cell carcinoma SAS cells. The sorted SP cells possessed much higher expression of stemness genes, such as *ABCG2*, *ABCC5*, *EpCAM*, *OCT-4*, *CD133*, *CD44*, and **β**-catenin, and more clonogenicity as compared with the Non-SP cells. After 48 h of treatment, honokiol dose dependently reduced the proportion of SP from 2.53% to 0.09%. Apoptosis of honokiol-treated SP cells was evidenced by increased annexin V staining and cleaved caspase-3 as well as decreased Survivin and Bcl-2. Mechanistically, honokiol inhibited the CD44 and Wnt/**β**-catenin signaling of SP cells. The Wnt signaling transducers such as **β**-catenin and TCF-4 were decreased in honokiol-treated SP cells, while the **β**-catenin degradation promoting kinase GSK-3**α**/**β** was increased. Consistently, the protein levels of **β**-catenin downstream targets such as *c-Myc* and *Cyclin D1* were also downregulated. Furthermore, the **β**-catenin-related EMT markers such as Slug and Snail were markedly suppressed by honokiol. Our findings indicate honokiol may be able to eliminate oral cancer stem cells through apoptosis induction, suppression of Wnt/**β**-catenin signaling, and inhibition of EMT.

## 1. Introduction

Oral squamous cell carcinoma (OSCC) is the most common type of head and neck cancer [1], which ranks the sixth worldwide for cancer-related mortality [2]. It accounts for almost 3% of cancer cases in the world [3]. In Taiwan, OSCC has the highest rate of increase among male cancers. Despite the improvements in surgical and radiotherapy, the five-year survival rate for oral cancer has remained unchanged at about 50% over the past 30 years [4]. Recently, targeted therapy has been useful primarily in early malignancies; however, it becomes ineffective eventually, due to the nonredundant tyrosine kinase receptors and activation of signaling through several pathways in advanced cancers [5]. As the available therapies are limited by the resistance and severe adverse side effects, more effective therapeutics are urgently needed to combat this life-threatening disease.

The cancer stem cell (CSC) hypothesis asserts that malignant tumors are maintained exclusively by a small subpopulation of cells that give rise to phenotypically diverse cancer cells [6–8]. In recent years, the CSC hypothesis has been studied in many types of cancers including OSCC, and there is growing evidence suggesting CSC is responsible for the tumor resistance to chemotherapy and radiation therapy [9, 10]. Targeting CSC has thus been a potential strategy to improve the treatment outcome of cancers.

The CSCs typically overexpress ATP-Binding cassette (ABC) transporters to pump out drugs and thus result in the resistance to chemotherapeutic agents [11]. This overexpression of ABC transporters could facilitate CSCs isolation by flow cytometry on the basis of their ability to pump out the fluorescent DNA binding dye such as Hoechst 33342. With the capacity to pump out the Hoechst 33342, the signals of CSCs fell to the “side” of the bulk positively stained cells in FACS (fluorescence activated cell sorter) analysis plots and were referred as side population (SP) cells [12]. The SP cells from OSCC have been shown to possess phenotypes of CSC such as elevated levels of ABCG2, CD44, Oct-4, and so forth, higher clonogenicity, and tumorigenicity of xenografts [1, 10]. We therefore used SP cells as a model to evaluate natural products with potential of targeting CSC.

Honokiol is an active component isolated from the bark of the traditional Chinese medicine *Magnolia officinalis*. It had been shown to exert various biological effects, including muscle relaxation, anti-inflammation, antioxidant activity, and various protecting effects against hepatotoxicity, neurotoxicity, thrombosis, and angiopathy [13]. Interestingly, honokiol had been reported to induce apoptosis or growth inhibition in a variety of cancer cell lines including breast, lung, colon, and prostate cancer [14–17] and inhibit tumor growth in xenograft animal models [14, 15, 17–21]. In head and neck cancer, the anticancer effects of honokiol also had been demonstrated both *in vitro* and *in vivo* [22, 23]. It is worthy and interesting to investigate if honokiol could eliminate the CSC in such type of cancer. Here, we studied the effects of honokiol on the cancer stem-like SP cells isolated from SAS human OSCC cells. In addition to apoptosis induction and CD44 surface marker expression, the Wnt/*β*-catenin signaling pathway, epithelial-mesenchymal transition (EMT) markers [24–26], and epithelial cell adhesion molecule (EpCAM) [27] which were known to play crucial roles in regulation of CSC properties were also examined.

## 2. Materials and Methods

### 2.1. Chemicals and Reagents

Honokiol (purity > 98%) was purchased from Sigma. Primary antibodies Survivin, Bcl-2, caspase-3, *β*-catenin, TCF-4, GSK-3*α*/*β*, c-Myc, Cyclin D1, CD44, Slug, Snail, and *β*-actin were purchased from Cell Signaling Technology. Honokiol was dissolved in dimethyl sulfoxide (DMSO) and further diluted in sterile culture medium immediately prior to use. The final concentrations of DMSO in cell cultures were all less than 0.05%. A TRIzol RNA isolation kit was obtained from Life Technologies, and primers for RT-PCR, dNTP, reverse transcriptase, and Taq polymerase were obtained from Gibco BRL. N2 supplement, human recombinant bFGF, and EGF were all purchased from Invitrogen.

### 2.2. Cell Culture

The human oral squamous cell carcinoma cell line SAS (JCRB0260) was purchased from the JCRB Cell Bank (Japanese Collection of Research Bioresources Cell Bank). This cell line was established from a poorly differentiated squamous cell carcinoma of the tongue. SAS oral cancer cells were maintained as monolayers in Dulbecco's modification Eagle's medium (DMEM) supplemented with 10% fetal bovine serum (HyClone) and 1% penicillin/streptomycin (Mediatech). Cells were cultured at 37°C in a water-jacketed 5% CO_2_ incubator.

### 2.3. Side Population Analysis and Purification Using Flow Cytometry

The side population (SP) cells were analyzed and sorted by Hoechst 33342 (Sigma) staining and FACSAria III cell sorter (BD). SAS cells were detached from the dishes with Trypsin-EDTA (Invitrogen) and suspended at 1 × 10^6^ cells/mL in Hank's balanced salt solution (HBSS) supplemented with 3% fetal calf serum and 10 mM HEPES. These cells were then incubated at 37°C for 90 minutes with 2.5 *μ*g/mL Hoechst 33342, either alone or in the presence of 50 *μ*M verapamil (Sigma), a nonspecific inhibitor of ATP-binding cassette transporter (ABC transporter). The diminishment of SP cells in the presence of verapamil was used to define the flow cytometry gate for sorting SP cells. After 90-minute incubation, the cells were centrifuged immediately for 5 minutes at 300 ×g, 4°C and resuspended in ice-cold HBSS. The cells were kept on the ice to inhibit efflux of Hoechst dye and 1 *μ*g/mL propidium iodide (BD) was then added to discriminate dead cells. Finally, these cells were filtered through a 40 *μ*m cell strainer (BD) to obtain single suspension cells for the analysis and sorting on FACSAria III flow cytometer.

### 2.4. Sphere Culture

After sorting, SAS side population cells were seeded with a density of 500 cells/well in 6-well ultra-low attachment plates (Corning) in DMEM/F-12 culture medium supplemented with N2 supplement, 10 ng/mL human recombinant bFGF, and 10 ng/mL EGF. After culture for 14 days, spheres were harvested for the subsequent assays of SP cells.

### 2.5. Colony Formation Assay

SAS SP or Non-SP cells were plated at about 200 cells per well in 6-well coated plates and cultured in the medium described in Section 2.4. for 10 days. After most cell clones had increased to >50 cells, they were washed with PBS, fixed in methanol for 15 min, and stained with crystal violet for 15 min at room temperature. After washing out the dye, colonies with >50 cells were counted as positive colonies. 

### 2.6. Assessment of the Proliferation of SAS SP and Non-SP Cells after Treatment with Honokiol

The SAS SP and Non-SP cells were seeded into 96-well plates in the medium described in Section 2.4. at a density of 3000 cells/well. After 24 h, the medium was replaced with fresh growth medium containing various doses of honokiol or vehicle only and the cells were incubated for another 48 hours. At harvest, cells were fixed with trichloroacetic acid (TCA) by gently adding it to each well to a final concentration of 10% with subsequent incubation for 1 hour at 4°C. The plates were then washed 5 times with tap water and air dried. The dried plates were stained with 100 *μ*L of 0.4% (w/v) Sulforhodamine B (SRB) prepared in 1% (v/v) acetic acid for 10 minutes at room temperature. The plates were rinsed quickly 4 times with 1% acetic acid to remove unbound dye and then air dried until no moisture was visible. The bound dye was solubilized in 20 mmol/L Tris base (100 *μ*L/well) for 5 minutes on a shaker. Optical densities were read on a microplate reader (Molecular Devices) at 562 nm. The optical density is directly proportional to the cell number over a wide range.

### 2.7. Flow Cytometric Assessment of Stemness Markers Expression

After being cultured in the medium described in Section 2.4. for 14 days, the SAS SP and Non-SP cells were harvested and incubated with phycoerythrin- (PE-) conjugated antibodies against Oct-4 (Santa Cruz), human CD133/2 (Miltenyi Biotec, recognizes epitope 2 of the CD133), and CD44 (Cell Signaling), respectively, at 4°C for 30 minutes. After washing the cells of excess reagents, the stained cells were immediately analyzed by a FACSCalibur flow cytometer (BD). Approximately 10,000 counts were made for each sample. Anti-IgG (isotype) were used to detect the non-specific binding background activity in this assay.

### 2.8. Flow Cytometric Assessment of Apoptosis Using Annexin V/PI Staining

Apoptosis was determined by using a commercially available Annexin V apoptosis detection kit (BD) and flow cytometry. The SAS SP cells were seeded in 6-well plate (2 × 10^5^ cells/well) in medium described in Section 2.4. and incubated for 24 h before treatment. Following treatment with different doses of honokiol for 48 h, the SAS SP cells were harvested, washed twice with 2 mL of ice-cold phosphate-buffered saline (PBS), and then incubated with 100 *μ*L of HEPES buffer containing 2 *μ*L of fluorescein-isothiocyanate-(FITC-) conjugated annexin V (10 *μ*g/mL) and 2 *μ*L of propidium iodide (PI, 100 *μ*g/mL) for 15 minutes. After washing the cells of excess reagents, 400 *μ*L of binding buffer was added. The stained cells were immediately analyzed with a FACSCalibur flow cytometer. Approximately 10,000 counts were made for each sample.

### 2.9. *β*-Catenin/Tcf Transcription Reporter Assay

SAS SP cells were plated in 6-well plates, grown to 80%–90% confluence, and transiently transfected with the plasmids of TOPflash. TOPflash has 3 copies of the Tcf/Lef binding sites in the upstream of a thymidine kinase (TK) promoter and the firefly luciferase gene. All transfections were performed with Lipofectamine and 1.8 *μ*g of TOPflash plasmids. To normalize transfection efficiency, cells were cotransfected with 0.2 *μ*g of the internal control reporter encoding *Renilla reniformis* luciferase driven under the TK promoter. After transfection, cells were incubated in the medium described in Section 2.4. with or without honokiol (0–10 *μ*M) for 48 h and then lysed with reporter lysis buffer at harvest. Luciferase activity was determined by using the Dual-Luciferase Assay System kit (Promega) according to the manufacturer's protocol. The experiments were performed in triplicate, and the results were expressed in relative luciferase units (RLU), normalized to transfection efficiency as described above. 

### 2.10. RT-PCR Analysis of Stemness Genes Expression

Trizol reagent was used to extract the mRNAs from the SAS SP and Non-SP cells according to the manufacturer's recommended protocol. Two *μ*g of RNA was added to RT-PCR reactions containing primers at a concentration of 0.5 *μ*M. After a 42°C/60 min reverse transcription step, 30 cycles of PCR amplification were performed at 94°C for 30 sec, 58°C for 50 sec, and 72°C for 50 sec. PCR products were run on 1.5% agarose gels for identification. Primers used were for *ABCG2*, forward: 5′-CATCAACTTTCCGGGGGTGA-3′ and reverse: 5′-TGTGAGATTGACCAACAGACCA-3′; for *ABCC5*, forward: 5′-CCAAGCTGACCCCCAAAATGAAAAA-3′ and reverse: 5′-TGGATGTGCTTGCCTTCTTCCTCTTC-3′; for *EpCAM*, forward: 5′-CTGCCAAATGTTTGGTGATG-3′ and reverse: 5′-ACGCGTTGTGATCTCCTTCT-3′; for *Oct-4*, forward: 5′-GGAGAGCAACTCCGATGG-3′ and reverse: 5′-TTGATGTCCTGGGACTCCTC-3′; for *CD133*, forward: 5′-CATAAAGCTGGACCCATTGG-3′ and reverse: 5′-CCTTGTCCTTGGTAGTGTTG-3′; for *CD44*, forward: 5′-CCTCCCTCCGTCTTAGGTCA-3′ and reverse: 5′-GGTAGCAGGGATTCTGTCTGT-3′; for **β*-catenin*, forward: 5′-ACTGGCAGCAACAGTCTTACC-3′ and reverse: 5′-TTTGAAGGCAGTCTGTCGTAAT-3′; for *GAPDH*, forward: 5′-ACCACAGTCCATGCCATCAC-3′ and reverse: 5′-TCCACCACCCTGTTGCTGTA-3′.

### 2.11. Western Blotting

After treatment with various concentration of honokiol for 48 h, the cell lysates were prepared using ReadyPrep Protein Extraction Kit (Bio-Rad) according to instructions provided. Total cell lysates (20 *μ*g) were separated electrophoretically by a 10% polyacrylamide SDS-PAGE gel and transferred onto a polyvinylidene fluoride membrane using the Bio-Rad Mini-Protean transfer system. The membrane was cropped and further incubated overnight at 4°C with specific antibodies against Survivin, Bcl-2, caspase-3, active *β*-catenin, TCF-4, GSK-3*α*/*β*, c-Myc, cyclin D1, CD44, Slug and Snail, and *β*-actin, respectively. After incubation with primary antibodies, the cropped membranes were washed with TBST 3 times and then incubated with horseradish peroxidase-labelled secondary antibody for 45 minutes at room temperature. After washing with TBST 3 times, final detection was performed with enhanced chemiluminescence (ECL) Western blotting reagents (Minipore) and BioSpectrum Imaging System (UVP, Upland, CA, USA).

### 2.12. Statistical Analysis

The significant difference between control and experimental groups was analysed using *t*-test (*P < 0.05; **P < 0.01).

## 3. Results

### 3.1. SAS SP Cells Possessed Characteristics of Cancer Stem Cell

**Figure 1:**
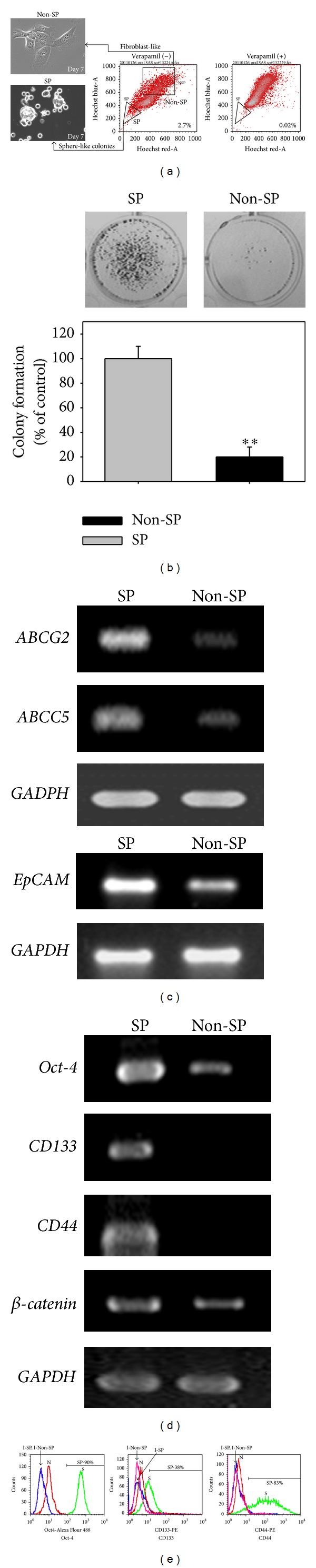
SAS SP cells possessed the properties of stem cells. (a) Spheroidal morphology of SP cells and fibroblast-like shape of Non-SP cells after 7 days of culture. The SP and Non-SP cells were sorted by Hoechst 33342 staining as indicated in the flow cytometry histogram and the gated region for dye excluding SP cells was identified by verapamil as described in Section 2. (b) SP cells had markedly higher clonogenicity than the Non-SP cells. The colonies formed were counted as described in materials and methods. (c) The mRNA expressions of multiple drug resistance genes (*ABCG2*, *ABCC5*) and epithelial cell adhesion molecule (*EpCAM*) in SP cells were much higher than those in Non-SP cells (*GAPDH* expression used as loading control). (d) The SP cells expressed much higher mRNA levels of stemness markers *Oct-4*, *CD133*, *CD44*, and **β**-*catenin* than the Non-SP cells (*GAPDH* expression used as loading control). (e) Flow cytometric analysis of stem cell markers expression in SP and Non-SP cells. The histograms display the expression of Oct-4, CD133, and CD44 in SP and Non-SP cells (I-SP, I-Non-SP: isotype control of SP and Non-SP cells, resp.; N: Non-SP cells; S: SP cells; the I-SP and I-Non-SP in the histograms of Oct-4 and CD44 are almost overlapped.).

To evaluate the effects of honokiol on the cancer stem cells, we sorted and characterized the SP cells from human SAS oral cancer cells as described. As shown in Figure 1(a), a small proportion (2.7%) of SP was detected in the left-down side of the flow cytometry histogram, which could be diminished in the presence of verapamil (50 *μ*M). After 7 days of culture, the sorted SP cells displayed sphere-like morphology in contrast to the flattened shape of Non-SP cells (Figure 1(a)). Besides, the SP cells appeared to have higher clonogenicity than the Non-SP cells (Figure 1(b)). In accordance with the lowered Hoechst blue-red intensity, the mRNA expression of ATP-binding cassette transporters such as ABCG2 and ABCC5 was much higher in SP cells as compared with that of Non-SP cells (Figure 1(c)). Recently, elevation of epithelial cell adhesion molecule (EpCAM) was reported to enhanced tumorsphere formation and tumor initiation [27]. Incompatible with this report, we found the SP cells also possessed much higher level of EpCAM mRNA than the Non-SP cells (lower part of Figure 1(c)). We next examined the expression of stemness markers in SP cells. As expected, the mRNA levels of Oct-4, CD133, and CD44 in SP cells were much higher than those of Non-SP cells (Figure 1(d)). This phenomenon was further confirmed by flow cytometry analysis, in which the peaks of SP cells markedly shifted apart from those of the Non-SP cells to the high intensity (right) side (Figure 1(e)). All these data indicated the CSC properties of SAS SP cells.

### 3.2. Honokiol Eliminated the SP Cells and Suppressed Their Clonogenicity

**Figure 2:**
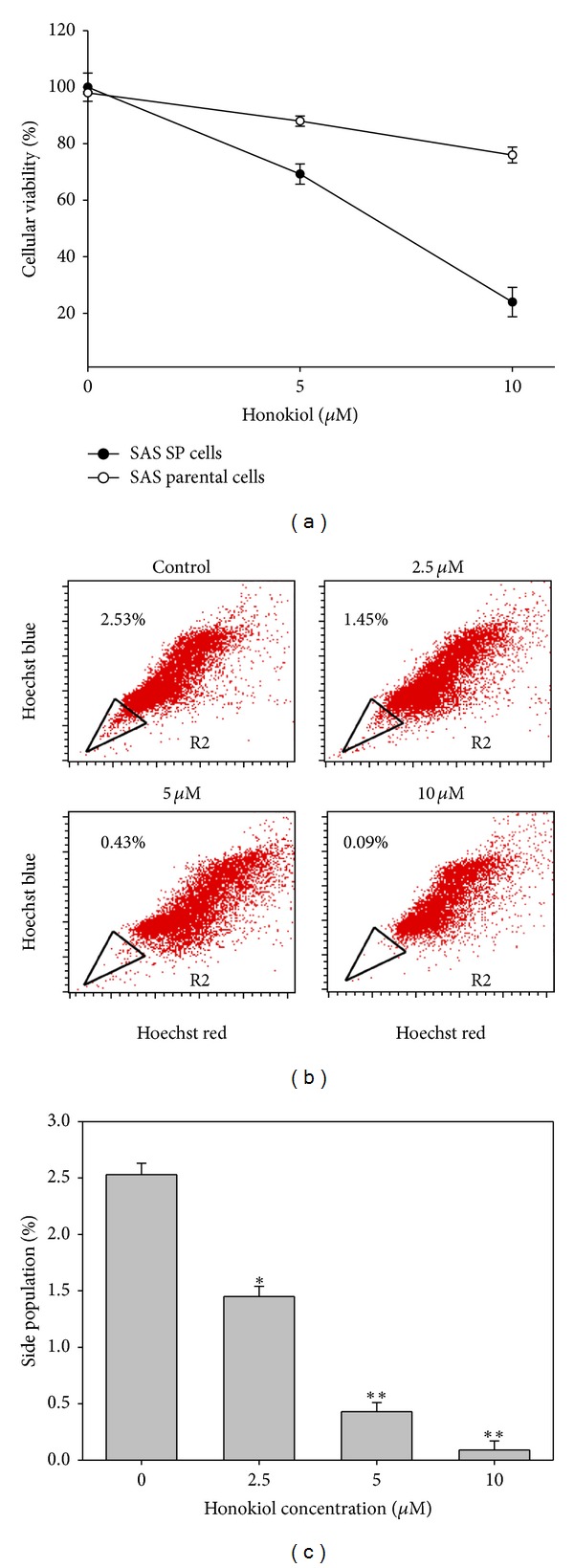
Effects of honokiol on SAS SP cells. (a) The honokiol-induced growth inhibition effects on SP and Non-SP cells after 48 h of treatment. (b) Honokiol decreased the proportion of SP in SAS cells after 48 h of treatment. The percentage of SP cells was indicated on the left-up side of the flow cytometric histograms. (c) Bar graph of the results shown in (b).

**Figure 3:**
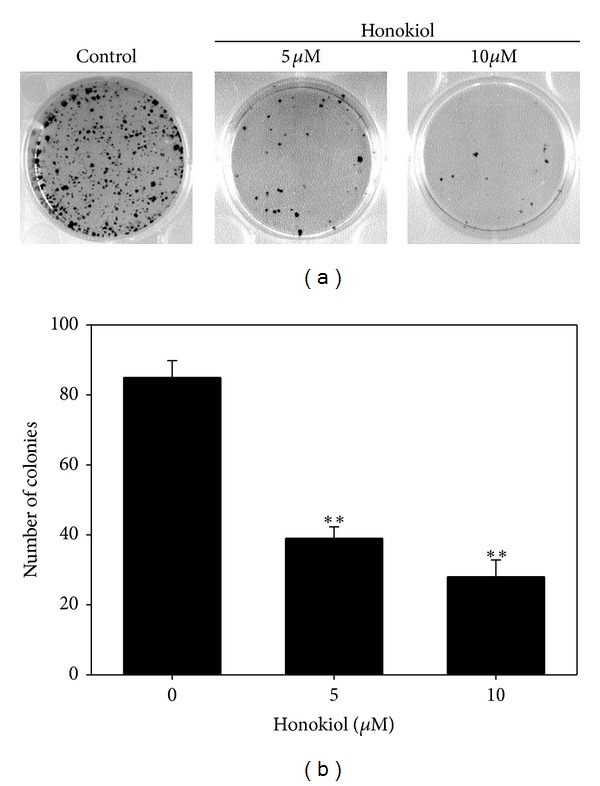
Effects of honokiol on the clonogenicity of SAS SP cells. (a) Honokiol markedly suppressed the colony formation of SAS SP cells in a dose-dependent manner. Cells were plated and then treated with honokiol for 10 days. The colonies formed were counted as described in Section 2. (b) Quantitative bar graph of the results shown in (a).

The sensitivities of SP and parental SAS cells to honokiol are quite different. The SP cells were markedly more sensitive than the parental SAS cells. After 48 h of treatment, honokiol suppressed 21% parental SAS cell growth at dose of 10 *μ*M. However, it inhibited 78% of SP cell growth at the same dose (Figure 2(a)). We then examined the percentage of SP in honokiol-treated SAS cells. After 48 h of incubation with honokiol at doses of 2.5, 5, and 10 *μ*M, the SP cells were dose dependently eliminated from 2.53% to 1.45%, 0.43, and 0.09%, respectively (Figures 2(b) and 2(c)). Accordingly, the clonogenicity of SAS SP cells was also markedly suppressed by honokiol at doses of 5 and 10 *μ*M (Figure 3).

### 3.3. Honokiol-Induced Apoptosis of SAS SP Cells

**Figure 4:**
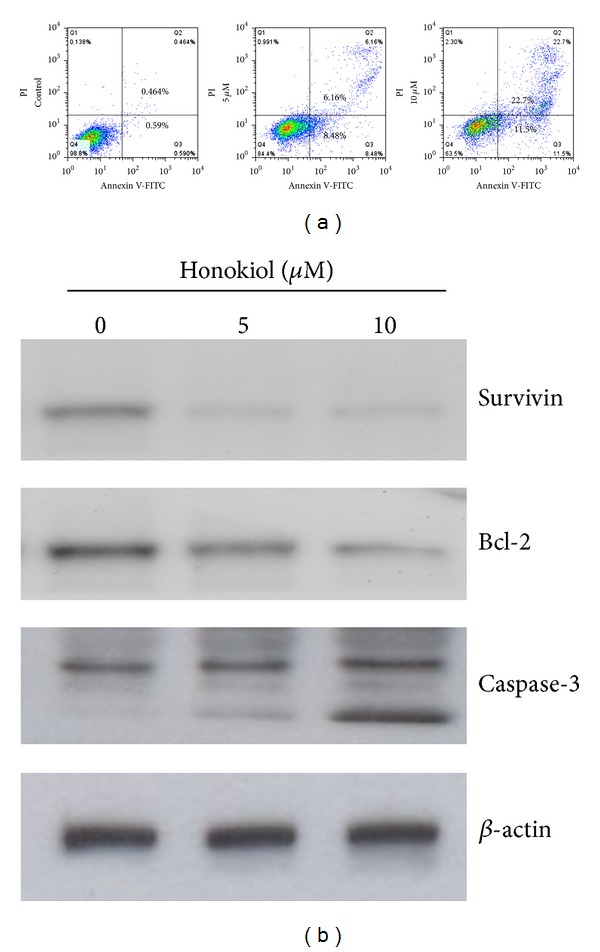
Honokiol-induced apoptosis of SAS SP cells. (a) Annexin V-PI staining of SP cells after treatment with honokiol for 48 h. Q1: quadrant (annexin V−, PI+) represents dead cells; Q2: quadrant (annexin V+, PI+) represents late apoptotic cells; Q3: quadrant (annexin V+, PI–) represents early apoptotic cells; Q4: quadrant (annexin V−, PI−) represents live cells. The percentage of cell population in each quadrant is calculated and shown in histograms and a dose-dependent increase of honokiol-induced apoptosis was displayed. (b) Decrease of Survivin and Bcl-2 protein levels and increase of caspase-3 cleavage in honokiol-treated SP cells. Cells were treated with honokiol for 48 h and the lysates were analyzed by Western blot using *β*-actin as loading control.

To investigate the mechanism of action underlying the SP cell elimination, we examined the apoptosis in honokiol-treated SP cells. After treatment with honokiol for 48 h, quantitative analysis of annexin V-FITC/PI staining by flow cytometry showed that the early apoptotic fraction (annexin V positive and PI negative) was increased from 0.59% of control to 8.48% and 11.5% at doses of 5 and 10 *μ*M, respectively. Simultaneously, the late apoptotic fraction (annexin V positive and PI positive) was increased from 0.46% of control to 6.16% and 22.7%, respectively (Figure 4(a)). This honokiol-induced apoptosis of SP cells was further confirmed by the dose-dependent cleavage of caspase-3 and downregulation of antiapoptosis proteins such as Bcl-2 and Survivin (Figure 4(b)).

### 3.4. Honokiol Inhibited the Wnt/*β*-catenin Signaling and EMT Markers in SAS SP Cells

**Figure 5:**
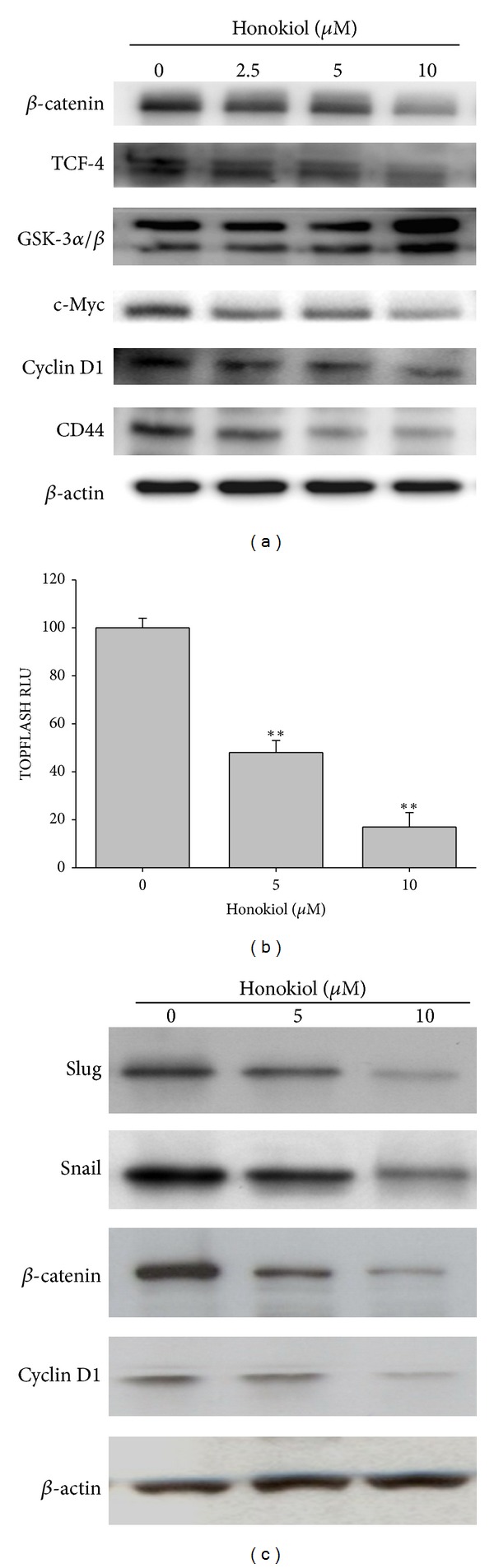
Honokiol downregulated the Wnt/*β*-catenin signaling and its downstream *in SAS SP cells*. (a) Honokiol decreased the protein levels of *β*-catenin and TCF-4 as well as their downstream c-Myc and Cyclin D1, accompanied with the increase of GSK-3*α*/*β* in SP cells. The expression of stem cell marker CD44 was also decreased. Cells were treated with honokiol for 48 h and the cell lysates were analyzed with Western blot using *β*-actin as loading control. (b) TOPFLASH TCF reporter assay in honokiol-treated SP cells. Cells were transfected with pTOPFLASH luciferase reporter constructs as described in Section 2 and treated with honokiol for 48 h, lysed, and analyzed using the Dual Luciferase Assay System kit. Results are expressed in relative luciferase units (RLU), normalized to transfection efficiency as described in Section 2. Each bar represents the mean of three independent transfections ±S.E. (c) Honokiol downregulated the EMT markers Slug and Snail proteins as well as Cyclin D1 while suppressed the *β*-catenin in SP cells. Cells were treated with honokiol for 48 h and then analyzed by Western blot as described in (a).

The Wnt/*β*-catenin pathway had been reported to play a crucial role in oral CSC and considered as an important target for screening therapeutics [26]. To more understand the molecular mechanisms responsible for the effects of honokiol against SAS SP cells, we analyzed the Wnt signaling pathway in honokiol-treated SP cells. Western blot results showed that honokiol not only decreased the Wnt signaling transducers such as *β*-catenin and TCF-4 (T cell transcription factor-4) but also increased GSK-3*α*/*β* (glycogen synthase kinase**)**, a *β*-catenin degradation promoting kinase. Consequently, the protein products of *β*-catenin downstream genes such as *c-Myc* and *Cyclin D1* were downregulated by honokiol (Figure 5(a)). Moreover, another putative CSC marker, CD44, was also inhibited as well (Figure 5(a)). To verify the inhibition on Wnt/*β*-catenin signaling pathway by honokiol, the TOPFLASH TCF reporter assay was performed for the activity measurement of Wnt/*β*-catenin signaling. Consistently, as shown in Figure 5(b), honokiol dose dependently inhibited the activity of Wnt/*β*-catenin signaling of SP cells.

Since it is known that Wnt/*β*-catenin pathway could mediate EMT (epithelial-mesenchymal transition), we thus examined the EMT markers in SP cells after treatment with honokiol. As shown in Figure 5(c), the protein levels of mesenchymal markers such as Slug and Snail were markedly suppressed, while *β*-catenin and its downstream Cyclin D1 were inhibited.

## 4. Discussion

OSCC is an important public health issue worldwide. To date, there is no effective treatment for patients with local relapse and distant metastasis. It remains a highly lethal and disfiguring disease [1, 28]. Recent studies showed that the resistance of OSCC to conventional chemotherapy or radiation therapy might be due to the presence of CSC [28]. Elimination of CSC would be an effective way to improve the clinical outcome of OSCC [10]. Searching the CSC targeting agents thus had been of great importance.

The “side population” (SP) is a well-known method to isolate cancer stem-like cells [12], although not all SP cells isolated from different cell lines possess CSC properties [29]. The SP from other OSCC cells had been found to possess CSC phenotypes and may play an important role in tumorigenesis [1]. Consistently, in this study, the SP isolated from SAS human OSCC cells was characterized with CSC properties as described. Moreover, Lin et al. recently reported that the expression of EpCAM in SAS OSCC cells was elevated when cultivated the attached cells into the tumorsphere [27]. In parallel with this, our results showed the mRNA level of *EpCAM* in SAS SP cells was much higher in comparison with that of Non-SP cells. Therefore, it is rational to use the SAS SP cells for investigating the effects of honokiol on CSC elimination.

According to recent studies, phytochemicals and herbs would be potential sources of therapeutics for CSC elimination. For example, resveratrol, curcumin, sulforaphane, and so forth had been reported to suppress the cancer stem-like cells [30]. Recently, Ponnurangam et al. found honokiol in combination with radiation could suppress the colonosphere formation and DCLK1^+^ and CD133^+^ populations of colon cancer cells, indicating the effects of honokiol on CSC inhibition [31]. In agreement with the study by Ponnurangam et al., we demonstrated the effects of honokiol on the elimination of CSC-like SP in OSCC cells. Ponnurangam et al. suggested honokiol targeted CSC by inhibiting the *γ*-secretase complex and the Notch signaling pathway in colon cancer [31]. In OSCC cells, our results showed honokiol suppressed the Wnt/*β*-catenin pathway and EMT markers of the CSC-like SP cells. The precise molecular mechanisms underlying the CSC targeting effects of honokiol may be complicated and variant in different types of cancer cells. Further investigation is needed to more precisely clarify the mechanisms of action.

The Wnt pathway is an important target for developing novel therapeutics targeting on CSC [26]. In OSCC cells, activation of Wnt pathway was shown to play a crucial role in the proliferation of CSC-like cells [28]. According to our results, the elimination of SP cells by honokiol may highly attribute to the inhibition of Wnt/*β*-catenin pathway, the most intensively studied and well-characterized Wnt pathway [26]. The level of *β*-catenin is tightly regulated by ubiquitin-proteasome-mediated degradation, which is controlled by a “destruction” complex composed of axin, adenomatous polyposis coli (APC), glycogen synthase kinase-3*β* (GSK-3*β*), and so on [26]. The *β*-catenin could be phosphorylated by GSK-3*β*, leading to its degradation. In response to Wnt, the GSK-3*β* will be inhibited, resulting in the accumulation of nonphosphorylated *β*-catenin, which could avoid degradation and translocate into the nucleus. In the nucleus, *β*-catenin forms a complex with T cell transcription factor (TCF) and other cofactors to activate the transcription of Wnt signaling target genes such as *c-Myc* and* Cyclin D1* [26, 32]. In accordance with these events of Wnt signaling, honokiol decreased *β*-catenin and TCF-4 of SP cells accompanied with the elevated GSK-3*α*/*β* and downregulated c-Myc and Cyclin D1 protein levels. The elevated GSK-3*α*/*β* might increase the formation of *β*-catenin/axin/GSK-3*β* destruction complex, a known precursor to *β*-catenin degradation. Based on our data, we deduce that the effects of honokiol on CSC elimination are highly related to Wnt signaling inhibition.

CD44, one of the well-known CSC markers, is initially used for breast CSC identification. Although CD44 alone is not sufficient for precisely isolating CSC in head and neck cancer cells, the CD44 expressing cells appear to have elevated tumorigenicity [10]. Consistently, our results showed the SAS SP cells expressed much higher level of CD44 than that of the Non-SP cells. Similar results were also observed in another OSCC cells by Zhang et al. [1]. As CD44 is also a target gene of Wnt signaling [33], the decreased CD44 in honokiol-treated SAS SP cells might also attribute to the inhibition of Wnt signaling cascade.

It is known that *β*-catenin could mediate epithelial to mesenchymal transition (EMT) [34], which plays a crucial role in cancer invasion and metastasis. The EMT markers such as Snail and Slug are also the target genes of *β*-catenin [35, 36]. Thus, the suppression of Snail and Slug in honokiol-treated SP cells might also result from the inhibition of Wnt/*β*-catenin signaling pathway. On the other hand, Mani et al. showed that EMT could generate cells with properties of stem cells [37]. Following this finding, extensive studies had demonstrated the link between EMT and CSC phenotype [38, 39]. Therefore, the suppressing effects of honokiol on the above EMT markers might also coincide with its effects against the stemness of CSC.

A number of nonsteroidal anti-inflammatory drugs (NSAIDs), such as aspirin, celecoxib, and sulindac, and natural compounds like epigallocatechin-3-gallate (EGCG), resveratrol, quercetin, curcumin, and so forth had been identified as inhibitors and/or modulators of Wnt/*β*-catenin signaling pathway [26]. Many of them such as EGCG, resveratrol, and curcumin were shown to have potential in CSC elimination [30]. Here, we demonstrated the substantial effects of honokiol on Wnt/*β*-catenin signaling inhibition and apoptosis induction in oral CSCs. As the biology of CSC is comprehensive and contains a considerable crosstalk in signaling pathways, combining honokiol with other CSC-eliminating agents listed above might provide better therapeutic effects. Further future studies to investigate these combination effects on CSC elimination are warranted.

## 5. Conclusions

Honokiol eliminated the CSC-like SP cells in SAS human oral squamous cell carcinoma cells. The underlying mechanisms were associated with apoptosis induction and the inhibition of Wnt/*β*-catenin cascade and related EMT markers. As CSC is a very important target for cancer therapy, our results further demonstrate the anticancer properties of honokiol and point out its potential application in CSC targeted therapy of oral cancer.

## References

[1] Zhang P, Zhang Y, Mao L, Zhang Z, Chen W (2009). Side population in oral squamous cell carcinoma possesses tumor stem cell phenotypes. *Cancer Letters*.

[2] Vermorken JB, Remenar E, van Herpen C (2007). Cisplatin, fluorouracil, and docetaxel in unresectable head and neck cancer. *The New England Journal of Medicine*.

[3] Silva SD, Ferlito A, Takes RP (2011). Advances and applications of oral cancer basic research. *Oral Oncology*.

[4] Chien HT, Liao CT, Huang SF (2011). Clinical significance of genome-wide minimally deleted regions in oral squamous cell carcinomas. *Genes Chromosomes & Cancer*.

[5] Arbiser JL (2007). Why targeted therapy hasn’t worked in advanced cancer. *The Journal of Clinical Investigation*.

[6] Reya T, Morrison SJ, Clarke MF, Weissman IL (2001). Stem cells, cancer, and cancer stem cells. *Nature*.

[7] Pardal R, Clarke MF, Morrison SJ (2003). Applying the principles of stem-cell biology to cancer. *Nature Reviews Cancer*.

[8] Beachy PA, Karhadkar SS, Berman DM (2004). Tissue repair and stem cell renewal in carcinogenesis. *Nature*.

[9] Clevers H (2011). The cancer stem cell: premises, promises and challenges. *Nature Medicine*.

[10] Monroe MM, Anderson EC, Clayburgh DR, Wong MH (2011). Cancer stem cells in head and neck squamous cell carcinoma. *Journal of Oncology*.

[11] Dean M, Fojo T, Bates S (2005). Tumour stem cells and drug resistance. *Nature Reviews Cancer*.

[12] Wu C, Alman BA (2008). Side population cells in human cancers. *Cancer Letters*.

[13] Fried LE, Arbiser JL (2009). Honokiol, a multifunctional antiangiogenic and antitumor agent. *Antioxidants & Redox Signaling*.

[14] Jiang QQ, Fan LY, Yang GL (2008). Improved therapeutic effectiveness by combining liposomal honokiol with cisplatin in lung cancer model. *BMC Cancer*.

[15] Wolf I, O’Kelly J, Wakimoto N (2007). Honokiol, a natural biphenyl, inhibits in vitro and in vivo growth of breast cancer through induction of apoptosis and cell cycle arrest. *International Journal of Oncology*.

[16] Hahm ER, Singh SV (2007). Honokiol causes G0-G1 phase cell cycle arrest in human prostate cancer cells in association with suppression of retinoblastoma protein level/phosphorylation and inhibition of E2F1 transcriptional activity. *Molecular Cancer Therapeutics*.

[17] Chen F, Wang T, Wu YF (2004). Honokiol: a potent chemotherapy candidate for human colorectal carcinoma. *World Journal of Gastroenterology*.

[18] Bai X, Cerimele F, Ushio-Fukai M (2003). Honokiol, a small molecular weight natural product, inhibits angiogenesis in vitro and tumor growth in vivo. *The Journal of Biological Chemistry*.

[19] Sheu ML, Liu SH, Lan KH (2007). Honokiol induces calpain-mediated glucose-regulated protein-94 cleavage and apoptosis in human gastric cancer cells and reduces tumor growth. *PLoS One*.

[20] Shigemura K, Arbiser JL, Sun SY (2007). Honokiol, a natural plant product, inhibits the bone metastatic growth of human prostate cancer cells. *Cancer*.

[21] Hahm ER, Arlotti JA, Marynowski SW, Singh SV (2008). Honokiol, a constituent of oriental medicinal herb Magnolia officinalis, inhibits growth of PC-3 xenografts in vivo in association with apoptosis induction. *Clinical Cancer Research*.

[22] Leeman-Neill RJ, Cai Q, Joyce SC (2010). Honokiol inhibits epidermal growth factor receptor signaling and enhances the antitumor effects of epidermal growth factor receptor inhibitors. *Clinical Cancer Research*.

[23] Chen XR, Lu R, Dan HX (2011). Honokiol: a promising small molecular weight natural agent for the growth inhibition of oral squamous cell carcinoma cells. *International Journal of Oral Science*.

[24] Chen C, Wei Y, Hummel M (2011). Evidence for epithelial-mesenchymal transition in cancer stem cells of head and neck squamous cell carcinoma. *PLoS One*.

[25] Bao B, Wang Z, Ali S (2011). Notch-1 induces epithelial-mesenchymal transition consistent with cancer stem cell phenotype in pancreatic cancer cells. *Cancer Letters*.

[26] Takahashi-Yanaga F, Kahn M (2010). Targeting Wnt signaling: can we safely eradicate cancer stem cells?. *Clinical Cancer Research*.

[27] Lin CW, Liao MY, Lin WW, Wang YP, Lu TY, Wu : HC (2012). Epithelial cell adhesion molecule regulates tumor initiation and tumorigenesis via activating reprogramming factors and epithelial-mesenchymal transition gene expression in colon cancer. *The Journal of Biological Chemistry*.

[28] Felthaus O, Ettl T, Gosau M (2011). Cancer stem cell-like cells from a single cell of oral squamous carcinoma cell lines. *Biochemical and Biophysical Research Communications*.

[29] Zhang H, Xi H, Cai A (2013). Not all side population cells contain cancer stem-like cells in human gastric cancer cell lines. *Digestive Diseases and Sciences*.

[30] Efferth T (2012). Stem cells, cancer stem-like cells, and natural products. *Planta Medica*.

[31] Ponnurangam S, Mammen JM, Ramalingam S (2012). Honokiol in combination with radiation targets notch signaling to inhibit colon cancer stem cells. *Molecular Cancer Therapeutics*.

[32] Ziegler S, Rohrs S, Tickenbrock L (2005). Novel target genes of the Wnt pathway and statistical insights into Wnt target promoter regulation. *The FEBS Journal*.

[33] Zeilstra J, Joosten SPJ, Dokter M, Verwiel E, Spaargaren M, Pals ST (2008). Deletion of the WNT target and cancer stem cell marker CD44 in Apc(Min/+) mice attenuates intestinal tumorigenesis. *Cancer Research*.

[34] Galichon P, Hertig A (2011). Epithelial to mesenchymal transition as a biomarker in renal fibrosis: are we ready for the bedside?. *Fibrogenesis & Tissue Repair*.

[35] Liu Y (2010). New insights into epithelial-mesenchymal transition in kidney fibrosis. *Journal of the American Society of Nephrology*.

[36] Conacci-Sorrell M, Simcha I, Ben-Yedidia T, Blechman J, Savagner P, Ben-Ze’Ev A (2003). Autoregulation of E-cadherin expression by cadherin-cadherin interactions: the roles of *β*-catenin signaling, Slug, and MAPK. *The Journal of Cell Biology*.

[37] Mani SA, Guo W, Liao MJ (2008). The epithelial-mesenchymal transition generates cells with properties of stem cells. *Cell*.

[38] Hayashida T, Jinno H, Kitagawa Y, Kitajima M (2011). Cooperation of cancer stem cell properties and epithelial-mesenchymal transition in the establishment of breast cancer metastasis. *Journal of Oncology*.

[39] Turner C, Kohandel M (2010). Investigating the link between epithelial-mesenchymal transition and the cancer stem cell phenotype: a mathematical approach. *Journal of Theoretical Biology*.

