# Corrigendum to “Honokiol Eliminates Human Oral Cancer Stem-Like Cells Accompanied with Suppression of Wnt/*β*-Catenin Signaling and Apoptosis Induction”

**DOI:** 10.1155/2017/9387837

**Published:** 2017-11-21

**Authors:** Chih-Jung Yao, Gi-Ming Lai, Chi-Tai Yeh, Ming-Tang Lai, Ping-Hsiao Shih, Wan-Ju Chao, Jacqueline Whang-Peng, Shuang-En Chuang, Tung-Yuan Lai

**Affiliations:** ^1^Department of Internal Medicine, School of Medicine, College of Medicine, Taipei Medical University, Taipei 11031, Taiwan; ^2^Center of Excellence for Cancer Research, Taipei Medical University, Taipei 11031, Taiwan; ^3^National Institute of Cancer Research, National Health Research Institutes, Miaoli 35053, Taiwan; ^4^Department of Surgery, Shuang Ho Hospital, Taipei Medical University, Taipei 23561, Taiwan; ^5^Graduate Institute of Clinical Medicine, Taipei Medical University, Taipei 11031, Taiwan; ^6^Graduate Institute of Medical Sciences, National Defense Medical Center, Taipei 11490, Taiwan; ^7^Department of Otolaryngology, Wan Fang Hospital, Taipei Medical University, Taipei 11696, Taiwan; ^8^Department of Traditional Medicine, Wan Fang Hospital, Taipei Medical University, Taipei 11696, Taiwan; ^9^Graduate Institute of Pharmacognosy, College of Pharmacy, Taipei Medical University, No. 250, Wu-Hsing Street, Taipei 11031, Taiwan

In the article titled “Honokiol Eliminates Human Oral Cancer Stem-Like Cells Accompanied with Suppression of Wnt/*β*-Catenin Signaling and Apoptosis Induction” [1], affiliation number one was incomplete. The corrected authors' list and affiliations are shown above.
